# The interlayer coupling modulation of a g-C_3_N_4_/WTe_2_ heterostructure for solar cell applications

**DOI:** 10.1039/d1ra08397j

**Published:** 2022-01-05

**Authors:** Peng Lin, Nengshen Xu, Xiaolin Tan, Xuhui Yang, Rui Xiong, Cuilian Wen, Bo Wu, Qilang Lin, Baisheng Sa

**Affiliations:** Key Laboratory of Eco-materials Advanced Technology, College of Materials Science and Engineering, Fuzhou University Fuzhou 350108 P. R. China linqilang@fzu.edu.cn bssa@fzu.edu.cn; College of Environmental Science and Engineering, Fujian Key Laboratory of Pollution Control & Resource Reuse, Fujian Normal University Fuzhou 350007 Fujian P. R. China xhyang@fjnu.edu.cn

## Abstract

Constructing van der Waals (vdW) heterostructures has been proved to be an excellent strategy to design or modulate the physical and chemical properties of 2D materials. Here, we investigated the electronic structures and solar cell performances of the g-C_3_N_4_/WTe_2_ heterostructure *via* first-principles calculations. It is highlighted that the g-C_3_N_4_/WTe_2_ heterostructure presents a type-II band edge alignment with a band gap of 1.24 eV and a corresponding visible light absorption coefficient of ∼10^6^ cm^−1^ scale. Interestingly, the band gap of the g-C_3_N_4_/WTe_2_ heterostructure could increase to 1.44 eV by enlarging the vdW gap to harvest more visible light energy. It is worth noting that the decreased band alignment difference resulting from tuning the vdW gap, leads to a promotion of the power conversion efficiency up to 17.68%. This work may provide theoretical insights into g-C_3_N_4_/WTe_2_ heterostructure-based next-generation solar cells, as well as a guide for tuning properties of vdW heterostructures.

## Abstract 2

g-C_3_N_4_/WTe_2_ heterostructure with tunable vdW gap shows a favorable solar energy conversion performance.

## Graphical abstract

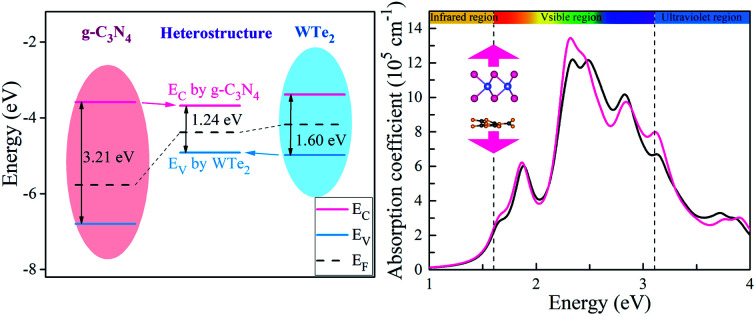

## Introduction

From graphene, two-dimensional (2D) materials open a new gate to the material society and provide us with unprecedented insight to understanding and exploring materials.^1,2^ Generally speaking, 2D materials could show distinguished physical and chemical properties due to their giant specific surface areas.^3^ For example, as the first discovered two-dimensional material, graphene has been demonstrated to be an outstanding candidate in tremendous applications such as Li-ion batteries, supercapacitors, and beyond.^4–6^ So far, the applications of various typical 2D materials have been investigated, involved in MXene, graphene-based materials, transition metal oxides, and so on.^7–10^ Besides, 2D materials present high performance not only in energy storages but also in catalysts, thermoelectric devices, electronic devices, and optoelectronic devices.^11–14^ Especially, many 2D semiconducting materials show dramatic light harvesting properties, inspiring global researchers to explore their applications in solar cells.^15^ Currently, the 2D transition metal dichalcogenides materials (TMDs) have been a research hotspot.^16,17^ TMDs are a class of materials with the formula MX_2_, where M is a transition metal element, and X presents for S, Se, and Te. These materials form layered structures with the X–M–X stacking configuration, where the chalcogens in two hexagonal planes are separated by a plane of transition metal atoms.^18^ The bulk TMDs have various properties ranging from insulators, semiconductors, semi-metals, and metals; meanwhile, their corresponding monolayers or few layers essentially preserve these properties.^19^ Multitudinous researches illustrated that TMDs could be a class of excellent materials in applications of photovoltaics and solar cells.^20^ On the other hand, the g-C_3_N_4_ and its isomers have been widely explored aiming at solar energy converting because of their high surface activities and easily modulated surface chemistry by means of surface engineerings.^21,22^ Monolayer g-C_3_N_4_ presents a suitable band gap leading to its favorable absorption properties in the visible light spectrum.^23,24^ However, the high recombination rate of electrons and holes in these individual 2D materials limits their performance in photocatalysts and solar cells.^25,26^ Hence, promoting the efficiency of carrier separations in 2D materials is of great interest and importance.^27,28^

Constructing van der Waals (vdW) heterostructures with different types of 2D materials stacking in a vertical direction has been proved an accessible approach to tune the properties and performance of 2D materials,^29–32^ which have been proved to be one of the most efficient categories to enhance the performance of TMDs and g-C_3_N_4_. It is noted that heterostructure solar cells, considered as next-generation solar cell technology, have attracted great attention because of their fascinating properties in solar cell application.^33,34^ For example, compared to single-layer structures, the optical properties under visible-light irradiation of Blue_P/TMDs vdW heterostructures are significantly improved combined, which achieves higher efficiency in solar energy conversions.^35^ Similarly, the g-C_3_N_4_ based heterostructures have tunable electric properties, stronger optical properties as well as higher catalytic activity.^36,37^ Especially, g-C_3_N_4_/WTe_2_ vdW heterostructure has been proved to be a potential electrocatalyst for hydrogen evolution reaction.^38^ At the same time, challenges and opportunities for exploring advanced g-C_3_N_4_ based heterostructure are still ongoing.

In this work, we investigated the interlayer interactions, electronic structures, and optical properties of an artificial g-C_3_N_4_/WTe_2_ vdW heterostructure. It is worth noting that vertical strains can modify the band gap and further result in a better light harvest with a light absorption coefficient up to ∼10^6^ cm^−1^ in the process. The decreased band alignment difference caused by the increased vdW gap gives rise to the promotion of power conversion efficiency are unraveled. Our findings provide significant guidance to design and modulate the performance of 2D materials applied in next-generation optoelectronic devices.

## Computational methods

In our work, we adopted the ALKEMIE platform^39^ together with the Vienna *ab initio* simulation package (VASP) based on density functional theory (DFT) to perform the first-principles calculations.^40^ The projection-augmented wave (PAW) exchange and correlation effects potential was used in the term of generalized gradient approximation (GGA) Perdew–Burke–Ernzerhof (PBE).^41–43^ We introduced the DFT-D3 method^44^ to correct the vdW interactions. A vacuum space of 20 Å along the *z*-direction was built to avoid periodic interactions. Energy cutoff of 500 eV was set, and 8 × 8 × 1 Γ-centered *k*-mesh was used for Brillouin zone (BZ) integrations. To overcome the underestimation of the band gap by the standard semilocal DFT functionals, we introduced the Heyd–Scuseria–Ernzerhof (HSE06) function^45^ for the electronic structure calculations. The relaxation convergence for electrons and ions were 1 × 10^−6^ eV and 1 × 10^−5^ eV, respectively. To obtain accurate dielectric functions comparable to the experimental results, time-dependent Hartree–Fock calculation (TDHF) was introduced to calculate the response functions by including the excitonic effects based on the HSE06 wavefunctions.

## Results and discussion

### Geometry and electronic structure

**Fig. 1:**
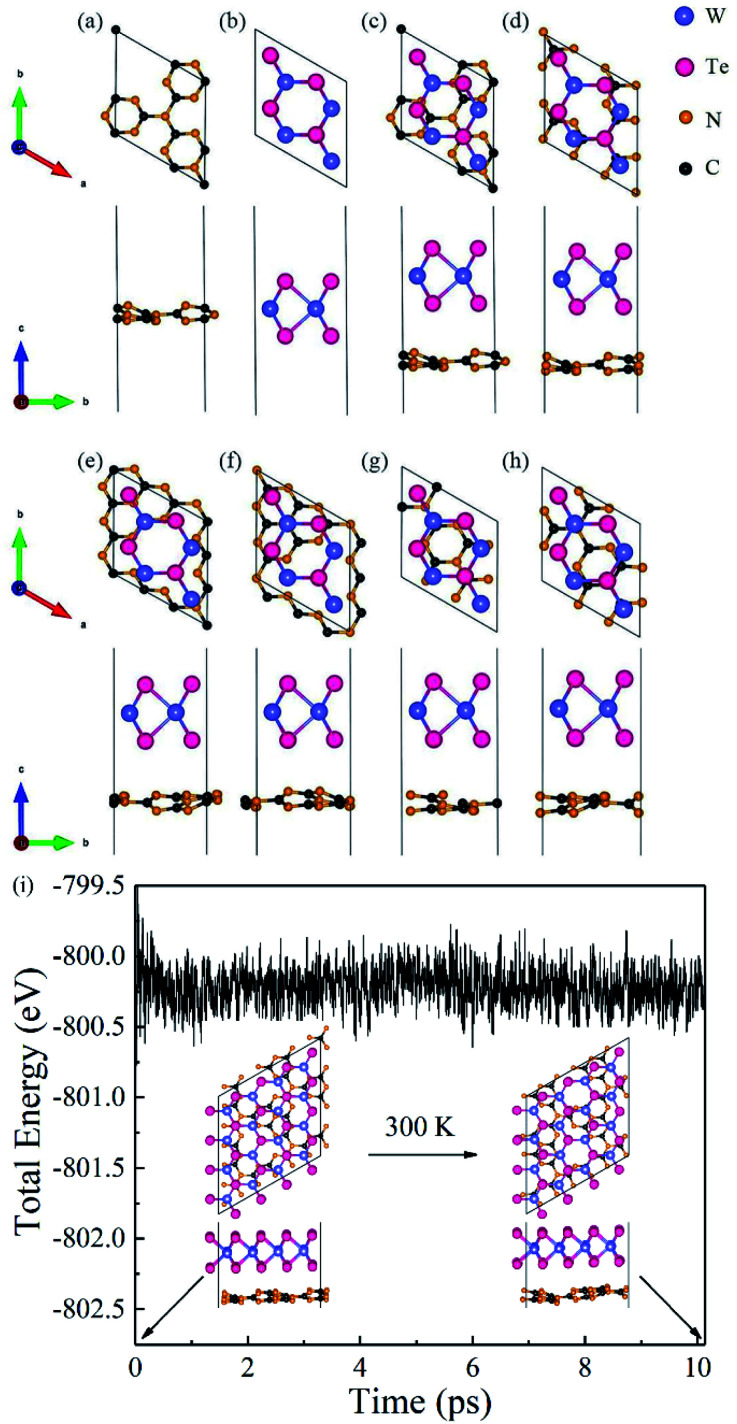
Top and side views for the structure of (a) g-C_3_N_4_ monolayer, (b) WTe_2_ monolayer, and (c–h) the six different stacking configurations of heterostructure. (i) The total energy changes and snapshots from AIMD calculations at 0 and 10 ps of g-C_3_N_4_/WTe_2_ heterostructure.

Firstly, we analyzed the geometry and electronic structures of monolayer g-C_3_N_4_ and WTe_2_. As shown in Fig. 1(a) and (b), g-C_3_N_4_ consists of N and C atoms in a staggered fashion similar to graphene with the optimized constant lattice of 6.95 Å, while monolayer WTe_2_ shows 2H phase with the optimized constant lattice of 3.52 Å, which agree well with previous works.^46,47^ We, therefore, built a g-C_3_N_4_/WTe_2_ heterostructure by stacking a 2 × 2 × 1 supercell of WTe_2_ upon the unit cell of g-C_3_N_4_ together with a lattice constant mismatch of 1.3%. Furthermore, we considered 6 possible stacking configurations by shifting g-C_3_N_4_ in a certain direction to explore the energetically favorable structure of the heterostructure, as illustrated in Fig. 1(c–h). Herein, the formation energy *E*_form_ was defined as1
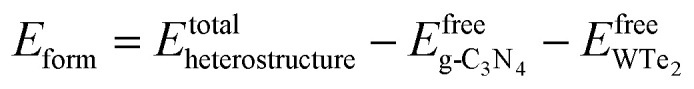
where *E*^total^_heterostructure_, 
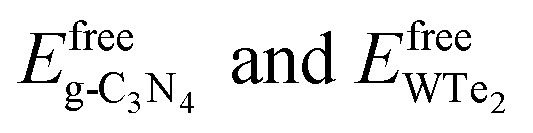
 are the total energy of the g-C_3_N_4_/WTe_2_ heterostructure, freestanding g-C_3_N_4_ and WTe_2_ monolayer, respectively. On the other hand, the vdW binding energy *E*_b_ was defined as^48^2
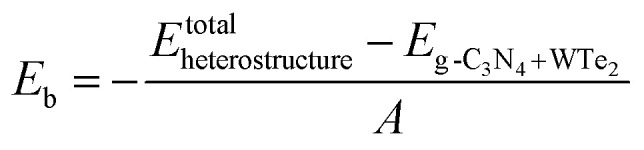
where *A* is the interface area of a heterostructure unit cell, *E*_g-C_3_N_4_+WTe_2__ is the sum of the total energies of the mutually independent g-C_3_N_4_ and WTe_2_ monolayers fixed in the corresponding heterostructure lattice, respectively. The optimized lattice constant *a*, the calculated vdW gap *d*_layer_, formation energy *E*_form_ and binding energy *E*_b_ are listed in Table 1. It is interesting to note that the values of *E*_form_ for all these 6 configurations are negative, indicating these heterostructures are energetic favorable. In addition, the calculated *E*_b_ between the g-C_3_N_4_ and WTe_2_ monolayers is around 15 meV Å^−2^, which is close to the typical vdW binding energy.^49,50^ Therefore, the g-C_3_N_4_/WTe_2_ heterostructure can be defined as a vdW heterostructure. We chose configuration-I as the object to study in the subsequent work since stacking configuration-I exhibits the most favorable *E*_form_ and smallest *d*_layer_.

**Table 1:** The calculated lattice constants *a*, the vdW gap *d*_layer_, formation energy *E*_form_ and binding energy *E*_b_ of g-C_3_N_4_/WTe_2_ heterostructure with possible stacking configurations

Configurations	I	II	III	IV	V	VI
*a* (Å)	6.993	7.098	6.989	6.988	6.993	6.986
*d*_layer_ (Å)	3.076	3.653	3.312	3.111	3.177	3.325
*E*_f_ (eV)	−0.652	−0.117	−0.645	−0.640	−0.604	−0.634
*E*_b_ (meV Å^−2^)	15.81	15.33	15.79	14.35	13.46	15.07

To prove the thermodynamically stability, Born–Oppenheimer *ab initio* molecular dynamics (AIMD) simulations were adopted for the proposed g-C_3_N_4_/WTe_2_ heterostructure at 300 K for 10 ps. A 2 × 2 supercell has been constructed for the AIMD calculations. Fig. 1(i) displays the energy evolution and structure snapshots after 300 K annealing for 10 ps of the g-C_3_N_4_/WTe_2_ heterostructures. It is noted that the structure snapshots suggest that atoms just move near their equilibrium location during the simulations, and there is no structural reconstruction at 300 K. At the same time, the changes of the total energy are very small during the simulations from Fig. 1(i), indicating that the proposed g-C_3_N_4_/WTe_2_ vdW heterostructure is thermodynamically stability at 300 K.

**Fig. 2:**
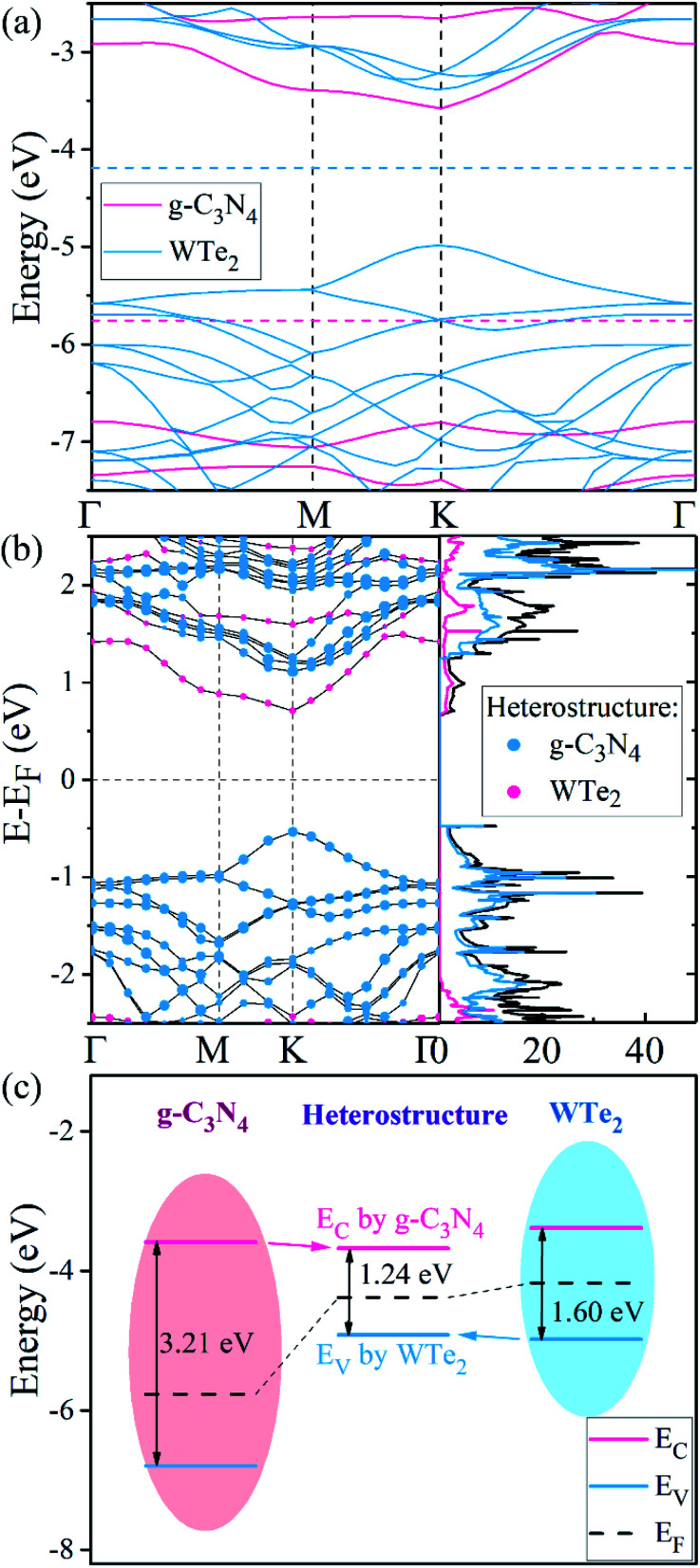
(a) The HSE06 band structure of free-standing g-C_3_N_4_ and WTe_2_ monolayer, respectively. (b) The projected HSE06 band structure and partial density of states of g-C_3_N_4_/WTe_2_ heterostructure. The size of pink and blue balls present the contributions from g-C_3_N_4_ and WTe_2_, respectively. (c) The band alignment diagrams for isolated g-C_3_N_4_, WTe_2_ monolayer and heterostructure interface.

Fig. 2(a) shows the band structures of freestanding g-C_3_N_4_ and WTe_2_ monolayers using HSE06 calculations. To compare clearly, the vacuum level was set to 0 eV as a baseline. It can be found that g-C_3_N_4_ has an indirect band gap of 3.21 eV, where CBM and VBM locate at the K (1/3, 1/3, 0) and Γ (0, 0, 0) point, respectively. Meanwhile, the WTe_2_ shows the direct gap feature with the band gap of 1.60 eV, where both CBM and VBM locate at the K (1/3, 1/3, 0) point. These results agree well with the previously published studies.^46,47^ On the other hand, the projected band structure and partial density of states of g-C_3_N_4_/WTe_2_ heterostructure is plotted in Fig. 2(b), in which the projected weight of g-C_3_N_4_ and WTe_2_ are distinguished by size and color. The pink and blue balls represent the contributions from g-C_3_N_4_ and WTe_2_, respectively. For g-C_3_N_4_/WTe_2_ heterostructure, both CBM and VBM locate at the K (1/3, 1/3, 0) point, showing the direct band gap feature, with the calculated HSE06 band gap of 1.24 eV. Interestingly, the g-C_3_N_4_/WTe_2_ heterostructure shows the band structure feature of a type-II heterostructure,^51^ where CBM is contributed by the g-C_3_N_4_ layer and VBM is occupied by the WTe_2_ layer. The band alignment diagrams for isolated g-C_3_N_4_, WTe_2_ monolayer, and heterostructure interface are illustrated in Fig. 2(c). Obviously, the work function of the g-C_3_N_4_/WTe_2_ heterostructure lies between the g-C_3_N_4_ and WTe_2_ monolayers. When g-C_3_N_4_ and WTe_2_ come into contact, the electrons flow from WTe_2_ to g-C_3_N_4_ due to the lower work function of WTe_2_ and *vice versa* for the holes. As a result of the increased transfer of electrons, the Fermi level shifts and finally reaches the same energy level. The differences between the band structure of g-C_3_N_4_/WTe_2_ heterostructure and corresponding monolayers indicate that the vdW interactions play an essential role in the electronic structures.

**Fig. 3:**
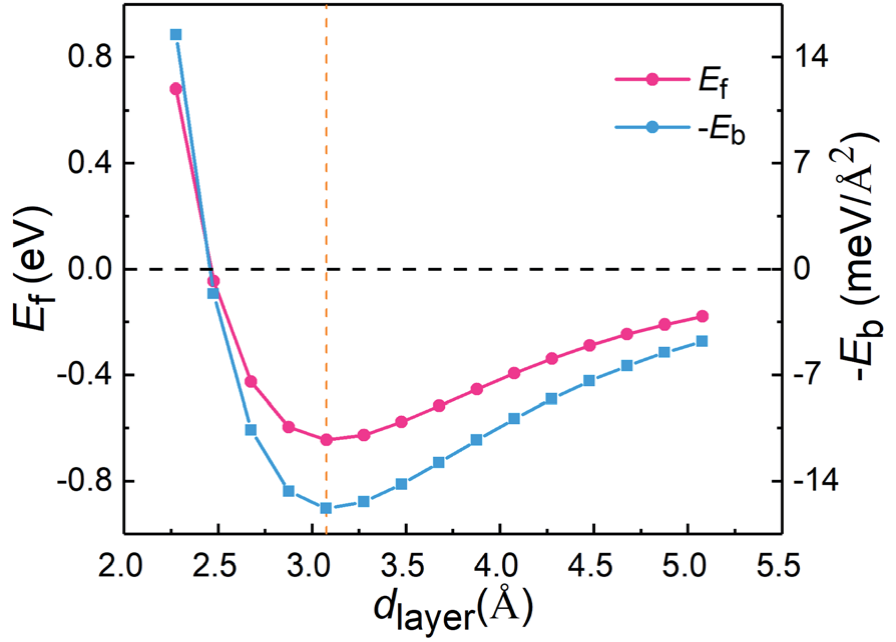
Formation energy *E*_form_ and binding energy −*E*_b_ as a function of interlayer distance *d*_layer_ of the vdW gap.

**Fig. 4:**
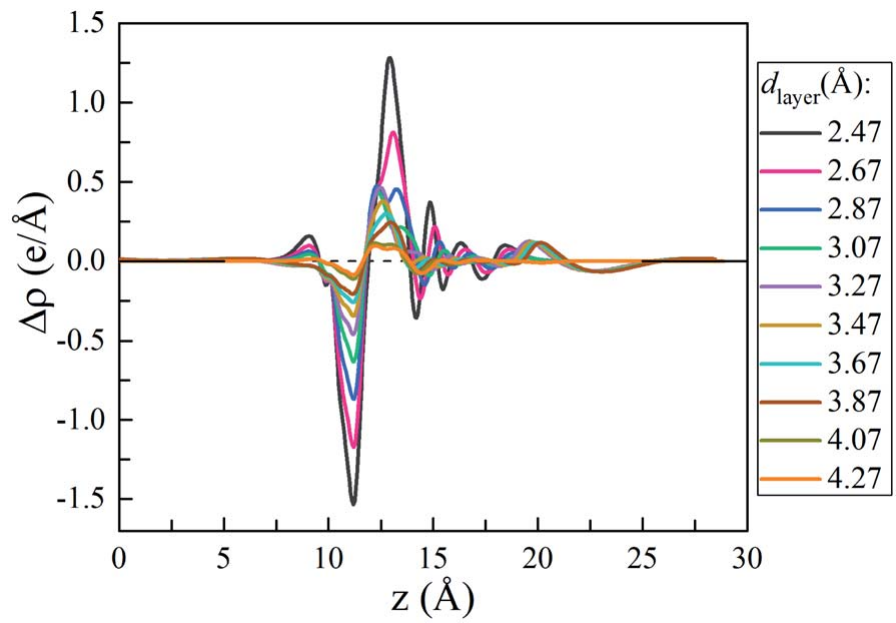
Plane-averaged charge density difference as a function of vdW gap.

To understand the vdW interlayer interaction between the different parts of the heterostructure, we further investigated g-C_3_N_4_/WTe_2_ heterostructure with different interlayer distance *d*_layer_ of the vdW gap. As shown in Fig. 3, both *E*_form_ and −*E*_b_ follow the Lenard-Jones type relation as a function of *d*_layer_,^52^ and a lower value of −*E*_b_ correspond to a stronger binding. Clearly, g-C_3_N_4_/WTe_2_ heterostructure with the equilibrium *d*_layer_ holds the most negative *E*_form_ and −*E*_b_. As the *d*_layer_ decreases, both *E*_form_ and −*E*_b_ increase dramatically. As the *d*_layer_ increases, *E*_form_ and −*E*_b_ gradually increases towards zero. Herein, *E*_form_ and −*E*_b_ remain negative among an extensive range of *d*_layer_, indicating the possibility to tune the interlayer interaction by varying *d*_layer_. As mentioned before, there is the transfer of electrons within the vdW gap, which affects the electronic structure of the g-C_3_N_4_/WTe_2_ heterostructure. Thereby, we calculated the planar-averaged charge density differences of g-C_3_N_4_/WTe_2_ heterostructure with different *d*_layer_, as shown in Fig. 4. Here, the plane-averaged electron density difference Δ*ρ* was calculated by

3 Δ*ρ* = *ρ*_g-C3N4/WTe2_ − *ρ*_g-C3N4_ − *ρ*_WTe2_

where *ρ*_g-C3N4/WTe2_ is the charge density of the heterostructure, *ρ*_g-C3N4_ and *ρ*_WTe2_ are charge densities of the g-C_3_N_4_ and WTe_2_ parts in the heterostructure, respectively. The positive and negative values denote charge accumulation and depletion in the combined system comparing with the two isolated monolayers, respectively. Fig. 4 clear presents the charge redistribution in the vdW gap of g-C_3_N_4_/WTe_2_ heterostructure: the charge depletion around the g-C_3_N_4_ part and the charge accumulation around the WTe_2_ region, indicating the charge transfer from g-C_3_N_4_ to WTe_2_. As the *d*_layer_ decreases, the stronger interlayer interaction results in the more obvious charge transfer. Oppositely, the charge transfer weakens when *d*_layer_ increases. The similar shape of Δ*ρ* for g-C_3_N_4_/WTe_2_ heterostructure with different *d*_layer_ indicates the excellent stability of the heterostructure from the electronic structure point of view. This phenomenon suggests a possible method to tune the band structure of the g-C_3_N_4_/WTe_2_ heterostructure by modifying the interlayer interaction.

**Fig. 5:**
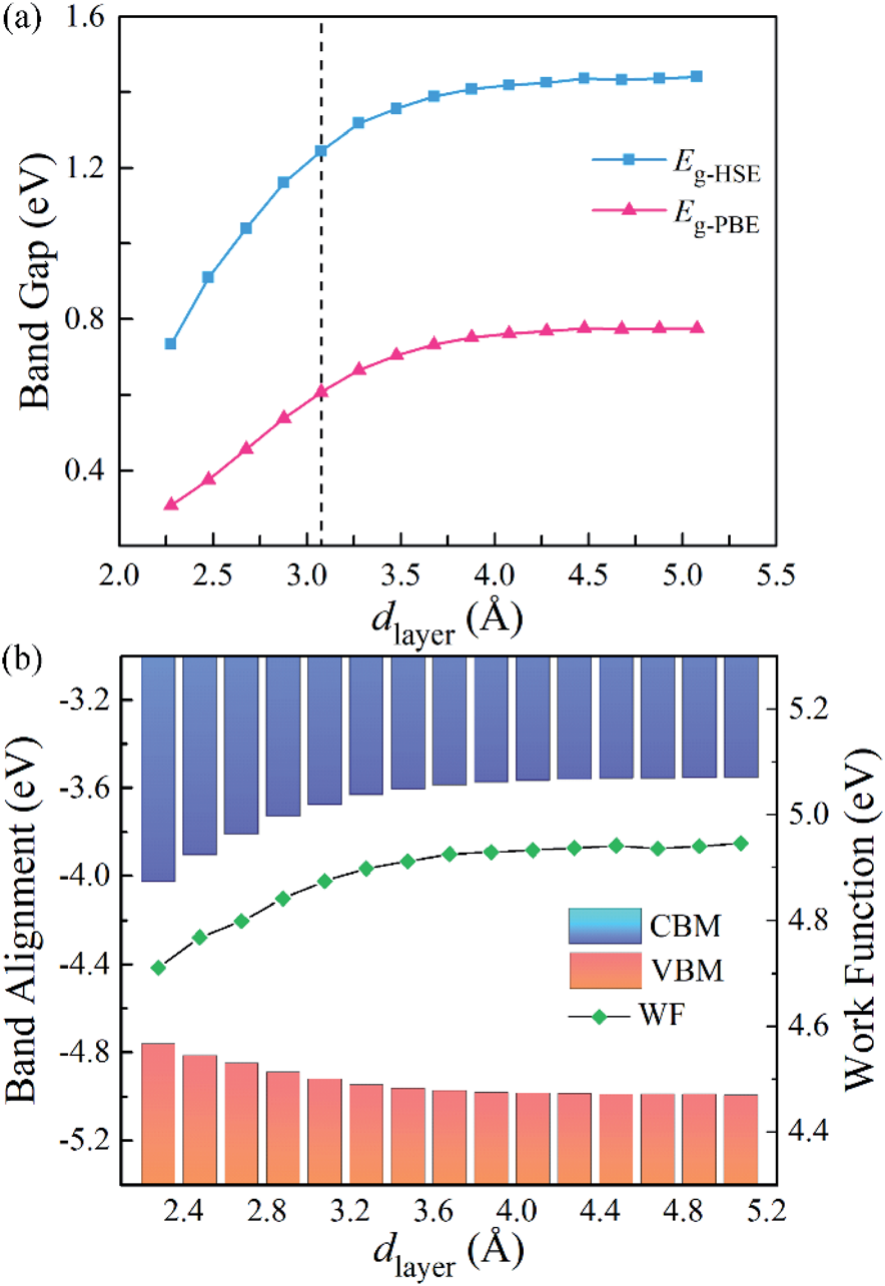
(a) Band gap, (b) band edge alignments and work function of g-C_3_N_4_/WTe_2_ heterostructure as a function of vdW gap.

To further explore the influence of the vdW interactions on the electronic structures of the g-C_3_N_4_/WTe_2_ heterostructure, we plotted the band gap, band alignment, and work function of g-C_3_N_4_/WTe_2_ heterostructure with different *d*_layer_ in Fig. 5(a). The PBE and HSE06 results show similar trends that the band gap decreases continuously as *d*_layer_ decreases. Oppositely, as *d*_layer_ increases, the band gap increases towards a balance value of 1.44 eV (HSE06). In addition, since the band alignment and work function are crucial in semiconductor heterostructure-based functional device designs, we plotted the band alignment and work function of the g-C_3_N_4_/WTe_2_ heterostructure corresponding to the vacuum level, as shown in Fig. 5(b). Correspondingly, the band alignment and work function show similar trends of band gap with different *d*_layer_. As the *d*_layer_ decreases, CBM shifts downward continuously, and VBM shifts upward continuously, which reduces the band gap. On the contrary, as *d*_layer_ increases, CBM and VBM shift oppositely and towards convergent.

**Fig. 6:**
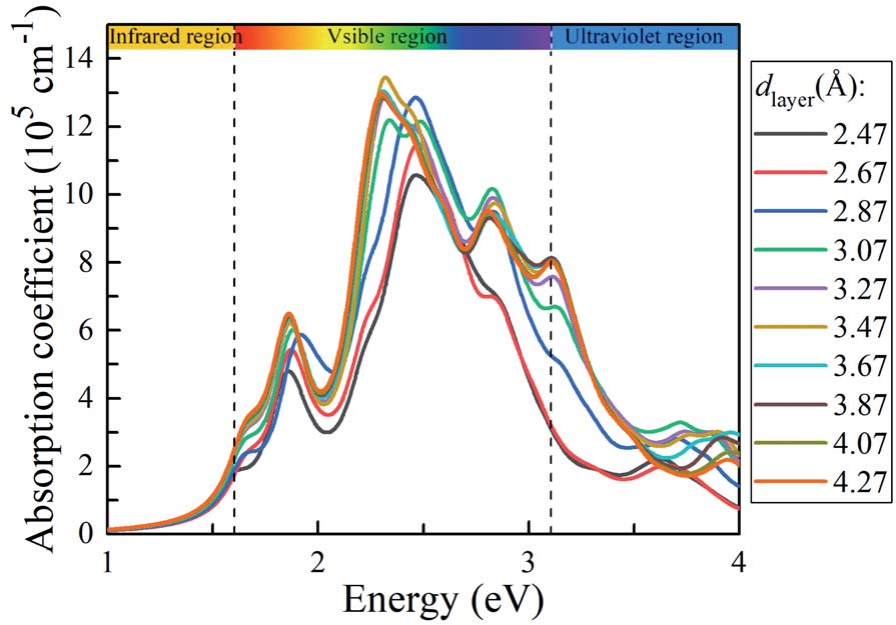
Absorption coefficient of g-C_3_N_4_/WTe_2_ with different vdW gap.

To explore the solar light-harvesting ability of the g-C_3_N_4_/WTe_2_ heterostructure, we calculated the optical absorption coefficients with a series of *d*_layer_. As presented in Fig. 6, there are three absorption peaks in the visible light region for the equilibrium vdW gap *d*_layer_ = 3.07 Å. The first absorption peak locates at ∼1.9 eV, and the main peak covers the light energy region of 2.25–2.6 eV with an ultra-high light absorption coefficient up to 1.22 × 10^6^ cm^−1^. And the third absorption peak locating at ∼2.8 eV presents the absorption coefficient of about ∼1 × 10^6^ cm^−1^. It is worth noting that the light-harvesting ability in the entire visible solar spectrum is elevated when the *d*_layer_ increases. Interestingly, the absorption peaks shift weakly towards the lower energy region as the *d*_layer_ rises, and the absorption coefficient increases the maximum value up to 1.34 × 10^6^ cm^−1^ when *d*_layer_ = 3.47 Å. Due to the direct band gap feature being beneficial for separating photo-excited electron–hole pairs and strong light absorption, the g-C_3_N_4_/WTe_2_ heterostructure could be a promising material for efficient photovoltaic solar cells and optoelectronic devices.

**Fig. 7:**
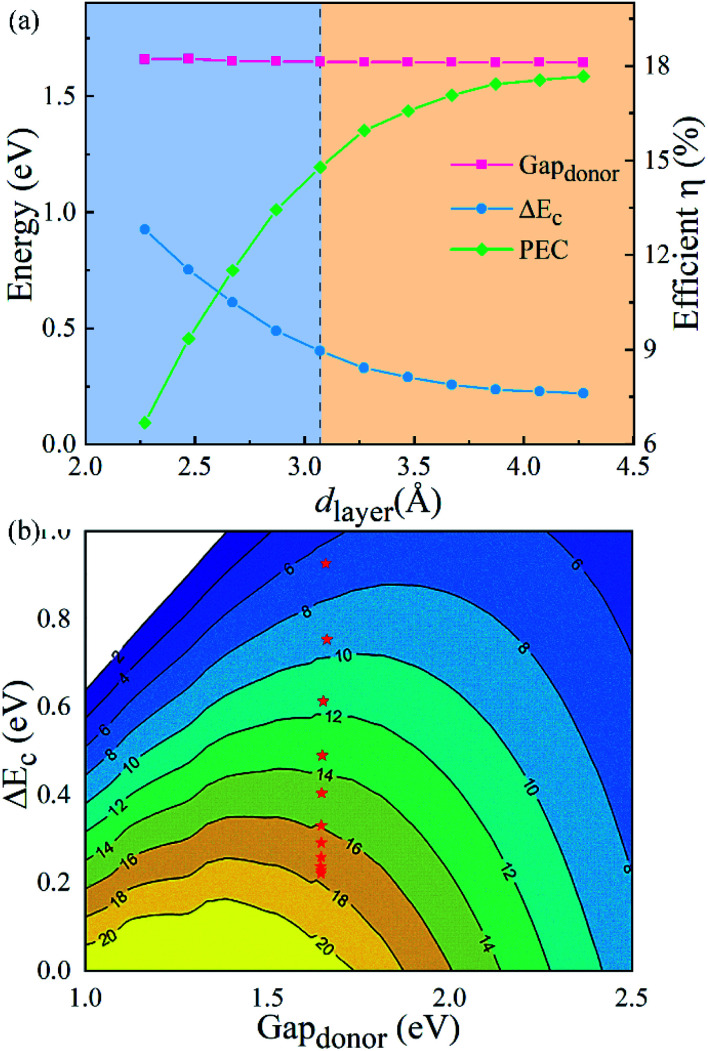
(a) The donor band gap Gap_donor_, conduction band offset Δ*E*_c_ and PCE of g-C_3_N_4_/WTe_2_ heterostructure as a function of vdW gap. (b) Contour plots for PCE as a function of the donor band gap and conduction band offset Δ*E*_c_.

Furthermore, we estimated the power conversion efficiency (PCE) by the method proposed by Scharber *et al.*,^53^ which is widely used in efficiency estimation. The upper limited PCE of the g-C_3_N_4_/WTe_2_ heterostructure is described by^54,55^4
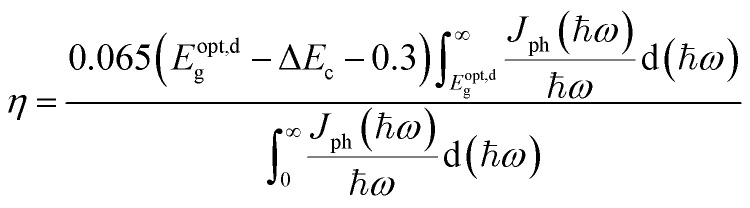
where 0.65 is the fill factor (*β*_FF_), *P*(ℏω) is the AM1.5 solar energy flux at the photon energy ℏ*ω*, *E*_g_ and Δ*E*_c_ are the band gaps of the donor and conduction band offset between donor and acceptor respectively. The (*E*^opt,d^_g_ − Δ*E*_c_ −0.3) term is an estimation of the open-circuit voltage (*V*_oc_). The integral term in the numerator is the short-circuit current density (*J*_sc_) assuming external quantum efficiency to be 100%, while the energy integral from 0 to infinity in the denominator is the power of incident solar radiation. Fig. 7(a) illustrates the donor band gap Gap_donor_ and conduction band offset Δ*E*_c_, which are critical to the maximum PCE, as well as simulated PCE of heterostructures with different *d*_layer_. Interestingly, due to a suitable band gap of about 1.4 eV with a Gap_donor_ of about 1.65 eV, the g-C_3_N_4_/WTe_2_ heterostructure shows an excellent solar spectrum absorption. Furthermore, the Gap_donor_ hardly changes, but the Δ*E*_c_ decreases about 70% in the process of compression and stretching. The reduced band offset differences in the stretching process lead to a higher PCE. Dramatically, the PCE improves considerably with a maximum value of 17.68% for the g-C_3_N_4_/WTe_2_ heterostructure. Fig. 7(b) depicts PCE variation with the Gap_donor_ and Δ*E*_c_. Therefore, we concluded that the g-C_3_N_4_/WTe_2_ heterostructure could show a better performance in solar cell applications by modifying the vdW gap.

## Conclusion

To conclude, based on the first-principle calculations, we have constructed the g-C_3_N_4_/WTe_2_ heterostructure and systematically analyzed the corresponding electronic band structure, optical properties with different *d*_layer_. As the *d*_layer_ increases, the band gap rises from 1.24 to 1.44 eV when the interlayer interactions become weaker, which brings an augmented light harvest in the visible range. Significantly, the maximum optical absorption coefficient can reach ∼10^6^ cm^−1^ level. Furthermore, the larger band gap and smaller band alignment difference make it better for light absorption and energy conversion. Finally, we found that the PCE of g-C_3_N_4_/WTe_2_ heterostructure has been promoted obviously during vdW gap tuning. The optimized PCE can reach up to 17.68%. Our results show that the g-C_3_N_4_/WTe_2_ heterostructure is favorable in solar cell applications. Here, we gave a tasteful way to realize the better performances of heterostructures, which is vital in the future study of vdW heterostructures.

## Conflicts of interest

The authors declare no competing financial interest.

## Supplementary Material
